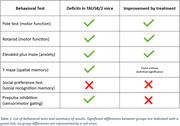# Therapeutic potential of chronic CBD:THC co‐treatment on disease‐relevant behaviors of female TAU58/2 mice

**DOI:** 10.1002/alz70859_099683

**Published:** 2025-12-25

**Authors:** Carla Lucia Wahlich, Tim Karl

**Affiliations:** ^1^ Friedrich‐Alexander‐University, Erlangen, Bavaria Germany; ^2^ School of Medicine, Western Sydney University, Campbelltown, NSW Australia

## Abstract

**Background:**

Limited therapeutic success and side effect profile of traditional but also novel antibody‐based therapies for Alzheimer's disease (AD) underline the need for alternatives. Cannabinoids have anti‐inflammatory effects, are easily accessible and generally well tolerated. A dosage‐dependent “entourage” effect has been described for phytocannabinoids such as cannabidiol (CBD) administered in combination with delta‐9‐tetrahydrocannabinol (THC). The effects of cannabinoid combination treatment on tau pathology, one of the major neuropathological hallmarks of AD, is poorly understood. Here, the effects of chronic treatment with CBD and THC on disease‐relevant behaviors of female TAU58/2 transgenic mice were evaluated for the first time.

**Method:**

Six‐month‐old TAU58/2 transgenic females (*n* = 28) and wild type‐like control littermates (*n* = 22) were chronically treated with CBD+THC (50:3 mg/kg/day, i.p.) or vehicle for five weeks. Behavioral testing started after three weeks of treatment and included assessment of motor function, spatial and social recognition memory, anxiety and sensorimotor gating.

**Result:**

Treatment and genotype effects on individual behavioral tests are summarized in Table 1. TAU58/2 transgenic females exhibited pronounced deficits in motor function, sensorimotor gating impairments, a prominent anxiolytic‐like phenotype and subtle spatial memory deficits. Chronic CBD:THC co‐treatment significantly improved aspects of motor function in pole test and accelerod. Moreover, anxiolytic‐like behavior of TAU58/2 mice was partially reduced by cannabinoid treatment. Cannabinoids also showed the potential to improve spatial memory impairment of transgenic mice, though not confirmed by a significant treatment effect. Social recognition memory and sensorimotor gating were not affected by the treatment.

**Conclusion:**

Here, long‐term CBD:THC treatment at 50:3 mg/kg/day shows subtle but promising therapeutic effects in middle‐aged TAU58/2 mice. Thereby, this study is the first to provide evidence for the therapeutic potential of CBD:THC co‐treatment on tauopathy‐related behavioral symptoms. Since CBD alone did not improve deficits of adult TAU58/2 mice in a previous study, these findings underline the potential of multi‐cannabinoid therapy for the treatment of AD and contribute to the evaluation of the most efficient cannabinoid ratio. Ongoing tissue analysis addressing tau and inflammatory markers will reveal further insights into the underlying molecular mechanisms.